# An Innovative Method for Sustainable Utilization of Blast-Furnace Slag in the Cleaner Production of One-Part Hybrid Cement Mortar

**DOI:** 10.3390/ma14195669

**Published:** 2021-09-29

**Authors:** Esraa K. Fayed, Fouad I. El-Hosiny, Ibrahim M. El-Kattan, Hussein Al-kroom, Mohamed Abd Elrahman, Hamdy A. Abdel-Gawwad

**Affiliations:** 1 Pyramids Higher Institution for Engineering and Technology, 6 October, Giza 12451, Egypt; esraakamal53@yahoo.com; 2 Chemistry Department, Faculty of Science, Ain Shams University, Cairo 12311, Egypt; 3 Environmental Science and Industrial Development Department, Faculty of Postgraduate Studies for Advanced Sciences, Beni-Suef University, Beni-Suef 18001, Egypt; ibrahemelkattan86@yahoo.com; 4 Civil Engineering Department, The University of Jordan, Amman 11942, Jordan; 5 Structural Engineering Department, Faculty of Engineering, Mansoura University, Mansoura 11001, Egypt; 6 Raw Building Materials and Processing Technology Research Institute, Housing and Building National Research Center (HBRC), 87 El-Tahreer St., Dokki, Giza, Cairo 12311, Egypt

**Keywords:** blast-furnace slag, sodium hydroxide, chabazite, activated species, compressive strength

## Abstract

Hybrid cement (HC) can be defined as alkali activated-blended-Portland cement (PC). It is prepared by the addition of an alkaline solution to high-volume aluminosilicate-blended-PC. Although this cement exhibits higher mechanical performance compared to conventional blended one (aluminosilicate–PC blend), it represents lower commercial viability because of the corrosive nature of alkaline solution. Therefore, this study focuses on the preparing one-part HC using dry activator–based BFS (DAS). DAS was prepared by mixing sodium hydroxide (NaOH) with BFS at low water to BFS ratio, followed by drying and grinding to yield DAS-powder. Different contents of DAS (equivalent to 70 wt.% BFS and 1, 2, and 3 wt.% NaOH) were blended with 30 wt.% PC. A mixture containing 70 wt.% BFS and 30 wt.% PC was used as a reference sample. The mortar was adjusted at a sand–powder (BFS-PC and/or DAS-PC) weight ratio of 3:1. The microstructural analysis proved that DAS-powder is mainly composed of sodium calcium aluminosilicate–activated species and unreacted BFS. These species can interact again with water to form calcium aluminum silicate hydrate (C-A-S-H) and NaOH, suggesting that the DAS acts as a NaOH-carrier. One-part HC mortars having 1, 2, and 3 wt.% NaOH recorded 7th day compressive strength values of 82%, 44%, and 27%, respectively, higher than that of the control sample. At 180 days of curing, a significant reduction in compressive strength was observed within the HC mortar having 3 wt.% NaOH. This could be attributed to the increase of Ca (within C-S-H) replacement by Na, forming a Na-rich phase with lower binding capacity. The main hydration products within HC are C-S-H, C-A-S-H, and chabazite as part of the zeolite family.

## 1. Introduction

Portland cement (PC) is a common and the dominant binding material in the construction sector [1,2]. About 8% of the total anthropogenic global warming potential is resulted from PC manufacturing [3]. Each metric tonne of PC requires 4.2 GJ energy, releasing approximately in 0.8–1.0 tonne of carbon footprint into surrounding environment [4]. To mitigate the high CO_2_ emission and energy demand, several authors replaced a high portion of PC by supplementary cementitious materials such as fly ash (FA), silica fume (SF), and blast-furnace slag (BFS) [5,6,7,8,9]. Although the role of supplementary cementitious materials in the mitigation of carbon footprint and the improvement of the durability and the later mechanical properties of PC have been established [10,11,12,13,14,15], the substitution of PC with a high volume of these materials caused a noticeable retardation in its early hydration [16,17,18].

BFS is simply defined as a calcium aluminosilicate-rich waste resulting from the top of smelted iron during the heating of iron ore in a blast furnace. The molten BFS was quenched with water to yield glassy materials with high amorphous content [19]. Statistically, the extraction of 1 tonne of iron from ore generates almost 0.3 to 1.0 tonne of BFS [20]. It is well known that BFS was used as a partial substituent to PC to yield what is called as slag cement [21]. Several authors have stated that the replacement of PC by high-volume BFS has reflected on a significant reduction of the heat of hydration, resulting in a retardation in the early compressive strength [22,23]. It was found that the performance and hydration characteristics of PC–BFS cement enhanced with increasing the curing temperature and the fineness of slag [24,25,26].

The addition of nano-silica led to a significant improvement in the early compressive strength accompanied by an acceleration in setting time of high-volume BFS blended cement [27,28,29,30]. A considerable improvement (16%) in the compressive strength of high-volume BFS blended cement was achieved by the addition of 1 wt.% nano-alumina [31]. The positive role of silica and alumina nano-particles is mainly originated from the formation of the additional calcium silicate hydrate (C-S-H), ettringite, and calcium aluminate hydrate (C-A-H), which have high efficiency in the mechanical properties improvement and the pore size reduction [27,28,29,30,31]. 

As an innovative approach, the addition of alkalis to high-volume BFS–PC has resulted in the formation of the alkali-activated BSF–PC, namely, hybrid cement (HC) [32,33]. The individual addition of sodium hydroxide (NaOH) and sodium silicate (Na_2_SiO_3_) to PC containing 80 wt.% produced hardened materials with compressive strength value 4.5 and 10.8 times, respectively, higher than that of the BFS-PC blend [34]. After dissolving alkali, it should be kept for enough time to cool before its mixing with the BFS-PC blend. The corrosive nature of alkaline solutions [35] is the common reason behind the retardation of the commercial viability of this type of cement. It is important to produce hybrid cement in which alkali is incorporated inside its composition. In other words, one-part HC (just added water needed) should be prepared to achieve the safe use of this type of cement. 

Therefore, this paper focused on the utilization of dry activator–based alkali-activated BFS (DAS) in preparing one-part hybrid alkali-activated slag-PC. DAS was prepared by mixing sodium hydroxide solution with BFS (at low water-to-powder ratio of 0.1), followed by drying and grinding to yield DAS-powder, which acts as a safe and stable NaOH-carrier. The ability of sodium leaching from DAS into surrounding media was evaluated using pH measurement. The impact of sodium oxide content within the dry activator on the performance of HC composed of 70 wt.% BFS and 30 wt.% PC was evaluated. This study also suggested the hydration reaction mechanism and the composition of strength-giving phases resulted from one-part HC. 

## 2. Experimental Program

### 2.1. Materials Resources

One-part hybrid cement (HC) mortar was fabricated from sand, ordinary Portland cement (OPC), blast-furnace slag (BFS), and sodium hydroxide (NaOH). Sand was brought from the El-Wasta area (Beni Suef, Egypt). OPC (type I: 42.5 N) was purchased from Beni-Suef Cement Company (Cairo, Egypt). BFS was supplied from Helwan Company for Steel Industry (Helwan, Egypt). NaOH with a purity of 99.99% was imparted by LOBA Chemical Company (Mumbai, India). Table 1 shows the chemical oxides compositions of OPC, BFS, and sand. As previously reported [36], BFS exhibits a completely amorphous pattern with a hump at 2theta of 20–35°. OPC and BFS demonstrated specific surface areas of 3410 and 3540 cm^2^/g, respectively. 

### 2.2. Preparation of One-Part Hybrid Cement Powder 

One-part hybrid cement powder was synthesized by mixing dry activator–based alkali-activated slag (DAS) with OPC. DAS was prepared by activating the BFS with NaOH solution, followed by drying and grinding. As shown in Table 2, BFS was individually activated by 1, 2, and 3 wt.% NaOH at W/BFS ratio of 0.1. The activated slurry immediately dried at 80 °C for 24 h, followed by grinding to yield DAS-powder. The W/BFS ratio of 0.1 was chosen according to a previously published work [37], which reported that W/BFS ratio of 0.1 is appropriate water content for the formation of alkali-activated powder with high capability to re-interact with water, yielding hardened materials. One-part HC was prepared by mixing DAS with OPC at different weights equivalent to 70 wt.% BFS and 1, 2, and 3% NaOH (by weight of BFS). A control sample containing 70 wt.% BFS and 30 wt.% OPC was made for comparison. The details of mixing proportions were listed in Table 3.

### 2.3. Preparation of One-Part HC Mortar

One-part HC mortar was designed at a sand–powder (BFS-OPC and/or DAS-OPC) weight ratio of 3:1. Sand and powder were dry mixed in a ball mill; after that, the dry blend was transferred to mixer, then the water was added (at W/P ratio of 0.47). Slow and rapid rates of wet mixing were applied on the fresh cement mortar to ensure complete homogeneity. The workable mortar was transferred into stainless steel molds with dimensions 50 × 50 × 50 mm^3^, followed by vibration, smoothing, and curing in relative humidity of 99 ± 1% at 23 ± 2 °C for 24 h. Thereafter, the hardened mortar was demolded and cured under tap water for 7, 28, 90, and 180 days. Cement paste with the same BFS-OPC and DAS-OPC weight ratios were prepared for investigating the hydration products. 

### 2.4. Experimental Methods

Different experimental methods, including flowability, setting time, zeta potential, and compressive strength, were carried out on the prepared one-part HC paste and/or mortar. The workability of the fresh one-part HC mortar and BFS-OPC mortar was determined by measuring the average spread diameters of the fresh mortar on flow table [38]. Initial and final setting times of the fresh pastes were conducted three times on each mixture using Vicat apparatus based on ASTM C191 [39]. Zeta potential of the fresh cement mortars was measured using Malvern Zetasizer (nano-series), in which deionized water was used as a carrier liquid. This test was conducted to determine electrostatic repulsion between hydrated cement particles within cement mortar. Compressive strength of the hardened one-part HC mortar was measured according ASTM C109M [40] using German-Bruf-Pressing Machine with a maximum load capacity of 175kN. This test was conducted on three hardened cubes of each mixture and the average reading was recorded. The broken paste was crushed and washed several times using acetone and methanol solution (at volume ratio of 1:1), then dried at 70 °C for 3 h, to stop the hydration reaction. After that, the dried paste was kept in a vessel until analyses. In contrast, the broken cement mortar was immersed in the same solution for 24 h, followed by drying at 70 °C for 3 h, and then kept until microstructural investigation. 

### 2.5. Instrumental Techniques

pH of the prepared DAS was conducted on the filtrate of the suspended solution (at DAS to distilled water weight ratio of 0.5) via Delta OHM HD 8705 pH meter and PCFC11 combination electrode with accuracy of 0.01. This test was conducted three times and the pH value was accepted if the variation rate of reading was less than 0.01/min. The oxides compositions of the starting materials were determined using X-ray fluorescence spectrometer (XRF: Xios, PW1400, Philips Company, Amsterdam, The Netherlands). The mineralogical compositions of the hardened cement pastes were identified using X-ray diffraction (XRD). XRD-analysis was conducted on the powdered-sample of the hydrated cement pastes using Philips PW3050/60 diffractometer with an X-ray source of Cu Kα radiation (λ = 1.5406 Å). The mineralogical compositions were determined within the 2theta range of 5–50° with 1s/step scanning rate and 0.05°/step resolution. Thermogravemetric analysis and its derivative (TG/DTG) was performed using DT-50 Thermal Analyzer (Schimadzu Co-Kyoto, Tokyo, Japan). This analysis was carried out on the powdered-cement paste to identify the hydration phases within its matrix. Each weight loss appeared at definite temperature is affiliated to the specific hydration product. This test was conducted by weighing 20 mg of sample in Pt-crucible, then heated in N2 atmosphere up to 1000 °C and heating rate of 10 °C/min. The functional groups within hydration products were identified using Fourier transform infrared (FT-IR) spectroscopy (KBr-discussing Genesis-II FT-IR spectrometer) at the wavelength range of 400–4000 cm^−1^. The microstructural development of the hardened one-part HC mortar was investigated using field emission scanning electron microscopy (FESEM, FEI Company, Holland) provided by an energy dispersive X-ray analyzer (EDS). 

## 3. Results and Discussion

### 3.1. Characterization of DAS

**Figure 1:**
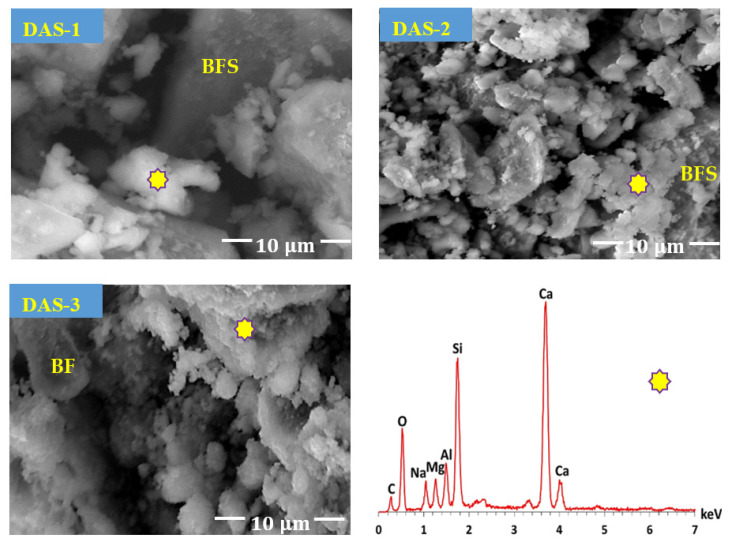
FESEM-micrographs and EDS-analysis of DAS-powders containing different NaOH contents.

The characterization of DAS is very important to determine its reaction mechanism and its role in the hydration of one-part HC. FESEM-micrographs (Figure 1) show that the addition of NaOH to BFS during preparing DAS has resulted in a partial dissolution of polygonal-shaped BFS particle to form sodium calcium aluminosilicate species as confirmed by EDS-analysis. Additionally, the formation of these species enhances with NaOH addition.

As stated by Davidovits [35], gehlenite and akermanite within BFS can be dissolved by NaOH to yield (Na, Ca)-ortho-sialate hydrate and calcium silicate hydrate as activated species. After that, these species condense together to form (Na, Ca)-cyclo-ortho-(sialate-disiloxo) and excess of calcium silicate hydrate. The polymerization process is the following step, in which a long chain of sodium calcium aluminosilicate is formed. The mixing water content plays an important role in the dissolution/condensation process. As previously stated [37], the use of low water during the activation process plays a circular role in the control of the condensation rate. The addition of low water content, followed by drying, causes a retardation in the condensation process, forming DAS with high activated species content. This is the main reason behind the justification of mixing water at W/BFS ratio of 0.1 and the application of drying during preparing DAS.

**Figure 2:**
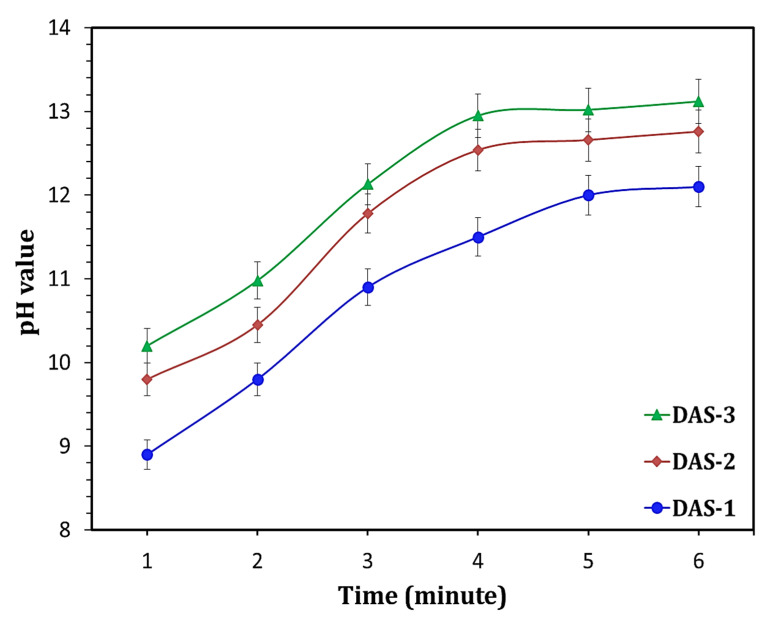
pH values of leachates of dry activator powders.

**Figure 3:**
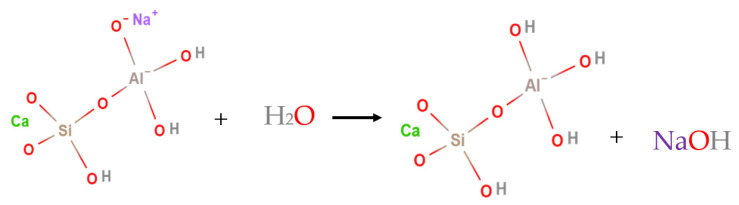
Interaction of activated species within dry activator powder with water.

To identify the reactivity of activated species, the leaching test was applied to the prepared DAS. As shown in Figure 2, a significant rising in pH-value in a short time (5 min) was recorded after suspension of DAS-powder in water. The possible explanation of this outcome is the moving Na cation at the ortho position within activated species, forming sodium hydroxide in the water medium (Figure 3). This means the high hydraulic reactivity of DAS, as the activation process can be continued after water addition. It can be said that the DAS acts as a carrier of NaOH, which strongly contributes to resolve the corrosive nature of alkali solutions during the preparation of one-part HC.

### 3.2. Flowability and Zeta Potential of One-Part HC Mortars

**Figure 4:**
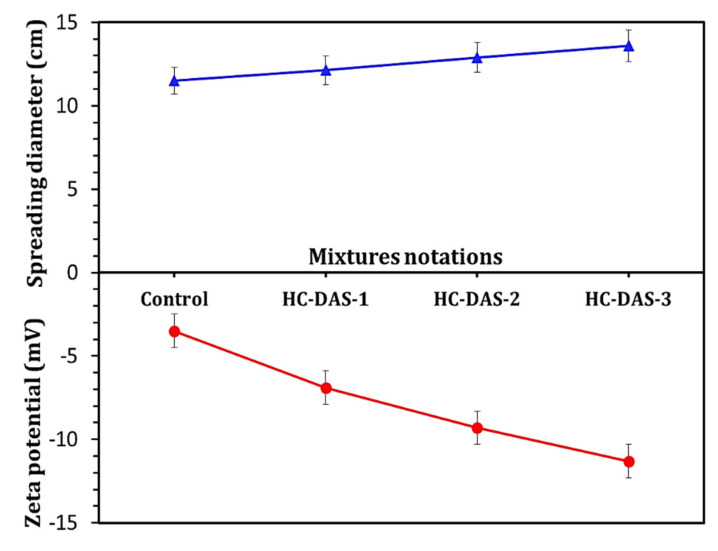
Workability and zeta potential of the fresh control and one-part HC mortars.

The impact of NaOH contents within DAS on the workability and zeta potential of fresh one-part HC mortar is represented in Figure 4. Generally, the spreading diameter of the fresh HC mortars indicates their workability. The fresh HC mortar with the broadest speeding diameter exhibits the best fluidity. There is a direct relationship between the flowability of the mortar and the negative zeta potential values. Increasing the NaOH content within DAS leads to a significant enhancement in the workability, which coincides with an increment in negative zeta potential value, as in line with the previously published work [41], which stated that the addition of Na_2_O content enhances the workability of one-part alkali-activated cement. He et al. [42] proved that increasing negative zeta potential is mainly originated from an increment in the electrostatic repulsive force between cement particles, resulting in an enhancement in the workability of the fresh cementitious material.

### 3.3. Setting Time of One-Part HC Pastes

**Figure 5:**
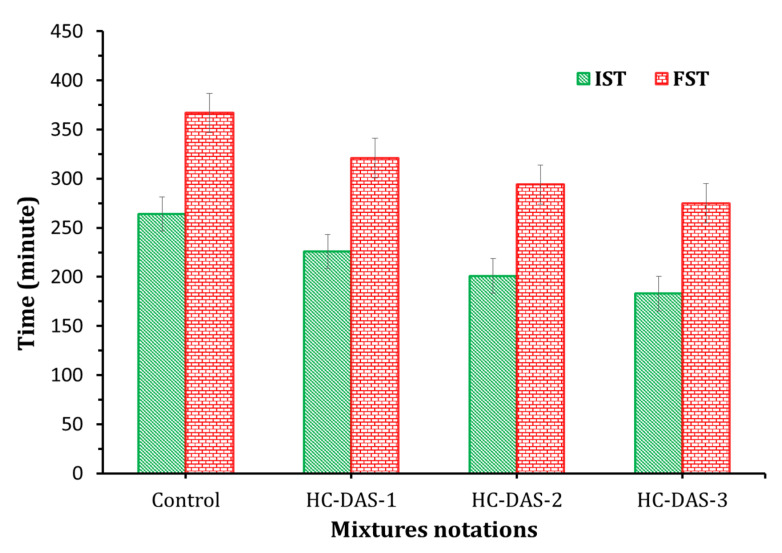
Setting time of the fresh BFS-OPC and one-part HC pastes.

The initial and final setting times (IST and FST, respectively) of the fresh BFS-OPC and DAS-OPC pastes are shown in Figure 5. The setting time of the fresh cement paste mainly depends on the content of NaOH within DAS. Depending on the composition of the fresh cement pastes, the IST was identified after 183–264 min; meanwhile, the FST was recorded after 275–367 min. One-part HC samples record shorter IST and FST compared to the BFS-OPC blend. The setting time decreases toward control > HC-DAS-1 > HC-DAS-2 > HC-DAS-3. This means that increasing NaOH content has a significant effect on the dissolution of aluminosilicate and the formation of binding phases [43]. 

### 3.4. Phase Identification

**Figure 6:**
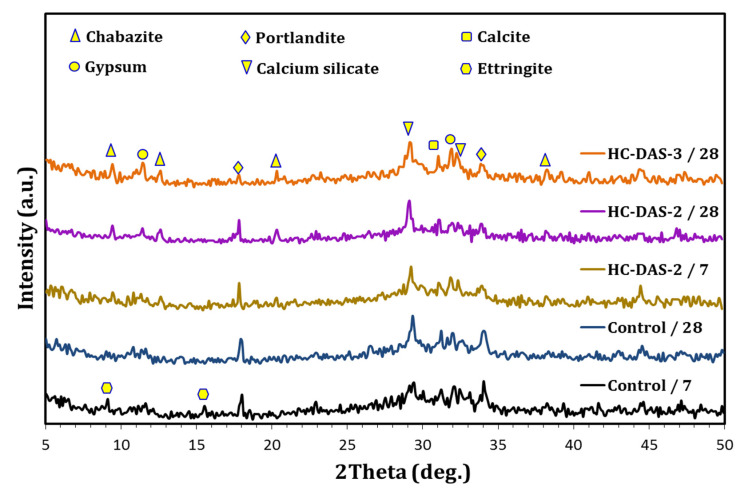
XRD patterns of the control sample and HC-DAS-2 pastes at 7 and 28 days as well as the HC-DAS-3 mixture at 28 days of curing.

The XRD-patterns of the BFS-PC blend (reference sample) and one-part HC pastes hydrated for 7 and 28 days are shown in Figure 6. Portlandite (Ca(OH)_2_), C-S-H, calcite, and ettringite are the main phases within control sample. Besides Ca(OH)_2_, C-S-H, and calcite peaks, chabazite (calcium sodium aluminum silicate hydrate: C-N-A-S-H) and gypsum were identified within the patterns referred to one-part HC pastes. For both control and one-part HC-DAS-2 mixtures, increasing curing times from 7 up to 28 days results in a significant depletion of Ca(OH)_2_ accompanied by an enhancement in C-S-H growth. This confirms the improvement of pozzolanic rate with time advanced, similar to the previous works [44,45,46]. Nevertheless, one-part HC-DAS-2 demonstrates the highest Ca(OH)_2_ consumption and C-S-H formation rates. Increasing NaOH content up to 3 wt.% within DAS has resulted in a significant increment in C-S-H formation at the expense of Ca(OH)_2_ phase. This confirms the fact that the activated aluminosilicate species within DAS can easily interact with Ca(OH)_2_ to yield C-S-H and/or C-N-A-S-H binding phases [47,48,49,50]. Ettringite has been detected within the pattern affiliated to control sample at 7 days of hydration. With the time advanced up to 28 days, the ettringite peaks disappeared. This confirms the dissociation of ettringite with curing time, as in agreement with previous reports [21,51]. On the other hand, the presence of alkali within one-part HC paste prevents the formation of ettringite. This could be the possible reason behind the appearance of gypsum within the hydrated one-part HC pastes. Halaweh [52] stated that the presence of alkali increases the rate of sulfate release into the solution, causing the instability of ettringite. 

**Figure 7:**
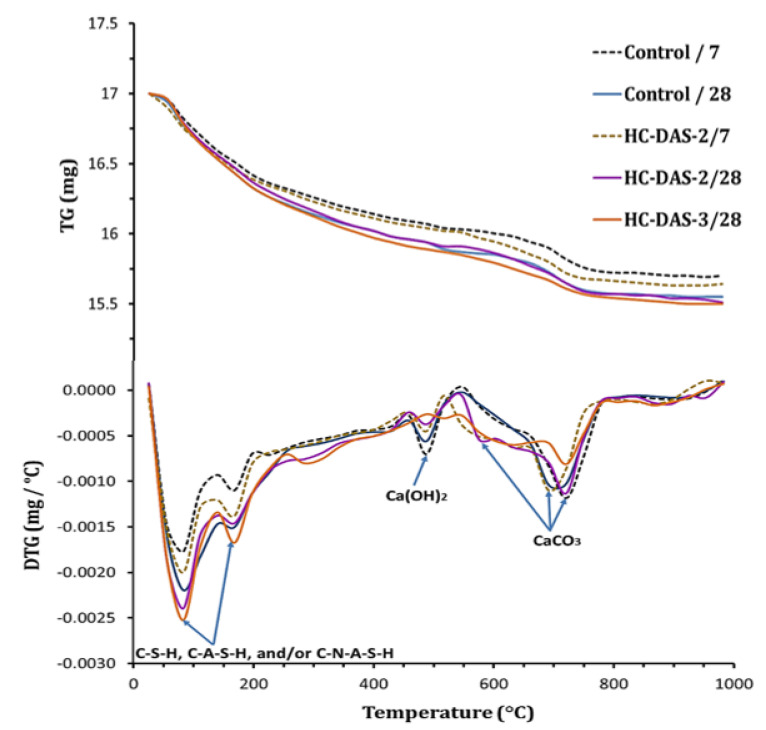
TG/DTG curves of the control sample and HC-DAS-2 pastes at 7 and 28 days as well as the HC-DAS-3 mixture at 28 days of curing.

Figure 7 represents the TG/DTG-curves of BFS-OPC blend and one-part HC hydrated for 7 and 28 days. All peaks are mainly related to the weight loss of the hydration phases within hardened pastes. The compositions of the hydration products strongly depend on the temperature at which the peak appears. The peaks that appear at the temperature range of 50–200 °C are mainly affiliated to the dehydration of combined water within C-S-H, C-A-S-H, and/or C-N-A-S-H [53,54]. Other peaks at 487 °C are related to the decomposition of Ca(OH)_2_ [55,56]. The peaks refer to the decarbonation of identified CaCO_3_ at a temperature range of 600–800 °C [57,58]. 

For BFS-OPC and one-part HC-DAS-2 mixtures, increasing curing time causes an enhancement in the intensity of C-S-H, C-A-S-H and/or C-N-A-S-H peaks at the expense of Ca(OH)_2_. This perfectly highlights the fact that the activation, hydration, and pozzolanic reactions are ongoing with time advanced. Comparing with the reference sample, the HC-DAS-2 mixture demonstrates higher C-S-H, C-A-S-H and/or C-N-A-S-H peaks intensity at 7 and 28 days of curing. This confirms the positive role of Na_2_O within DAS in the acceleration of the pozzolanic reaction and the formation of excessive binding-phases content. As aforementioned, the DAS is mainly composed of reactive sodium calcium aluminosilicate species. These activated species can hydrate and interact with Ca(OH)_2_ to yield binding phases easier than gehlenite and akermanite mineral within BFS. Increasing NaOH content up to 3 wt.% (HC-DAS-3) enhance the formation of C-S-H, C-A-S-H, and/or C-N-A-S-H accompanied by a significant consumption of Ca(OH)_2_. These outcomes agree with the XRD results. 

### 3.5. Compressive Strength

**Figure 8:**
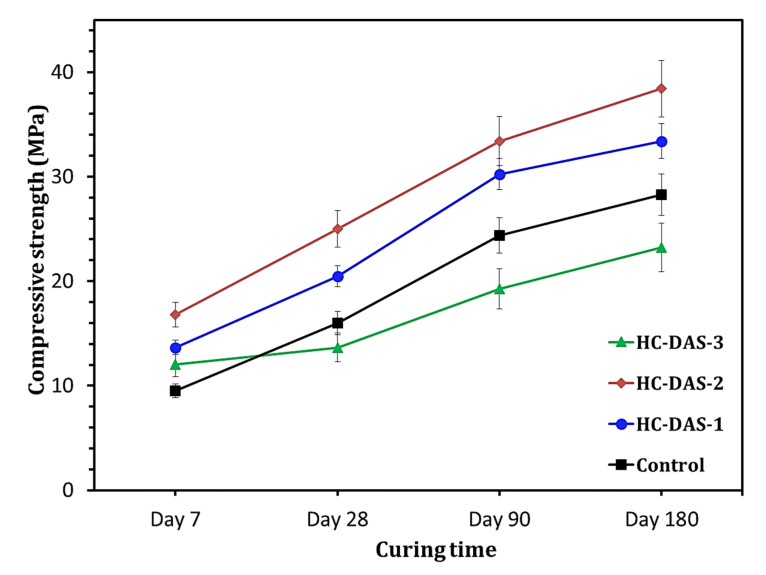
Compressive strength of the hardened BFS-OPC blend and one-part HC mortars.

The compressive strength values of the hardened BFS-OPC and one-part HC mortars are graphically represented in Figure 8. Increasing curing time (up to 180 days) is found to have a positive impact on the compressive strength development. This means the successive formation of strength-giving phases (C-S-H C-A-S-H, and/or C-N-A-S-H) with time advanced as confirmed by XRD and TG/DTG analyses. The same trend is observed in the previously published reports [33,59,60]. Increasing NaOH content within DAS up to 2 wt.% materially improves the early compressive strength (at 7 days). The NaOH-content beyond 2 wt.% reduces the 7th day compressive strength, but it is still higher than that of the control sample (BFS-OPC). One-part HC mortars individually containing DAS-1, DAS-2, and DAS-3 exhibit 7th day compressive strength of ~44%, 77%, and 27%, respectively, higher than that of the hardened BFS-OPC mortar. At later ages (28 to 180 days), the HC-DAS-1 and HC-DAS-2 show the same trend. The hardened HC-DAS-3 achieves later compressive strengths lower than those of the control sample. This proves the fact that the NaOH-content released from DAS (Figure 5) plays an important role in the performance of one-part HC mortar. 

Cement hydration and alkali BFS activation are the two synergistic mechanisms of one-part HC mortar. The activated aluminosilicate-species-containing DAS interacts with water to yield C-A-S-H and NaOH (Figure 5). The unreacted BFS within DAS is dissolved by the liberated NaOH through the alkali-activation process, yielding C-N-A-S-H, as confirmed by XRD analysis (Figure 8). On the other hand, the OPC within one-part HC mortar also interacts with water to produce C-S-H and Ca(OH)_2_. An additional C-N-A-S-H could be formed through the consumption of Ca(OH)_2_ by the activated species resulted from alkali BFS-activation. In contrast, the formation of strength-giving phases is resulted from the pozzolanic reaction, as C-S-H and/or C-A-S-H are formed through the interaction between aluminosilicate within BFS and Ca(OH)_2_ resulted from OPC hydration.

**Figure 9:**
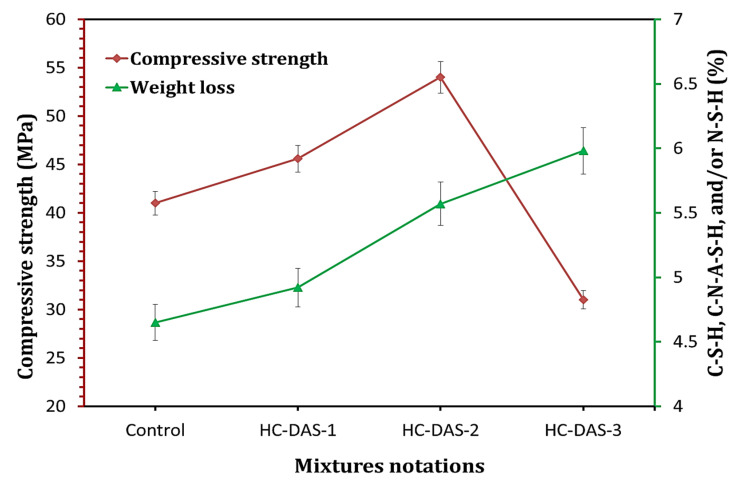
Relationship between weight losses of the hydration products within the hardened cement pastes and the compressive strength.

It is recognized that the enhancement of hydration products’ content strongly reflects on the mechanical properties of the hydrated cement [37,41,53,61,62]. Accordingly, a relationship between compressive strength and hydration products content (determined by TG analysis) within cement pastes hydrated for 28 days is represented in Figure 9. Increasing NaOH content (up to 2 wt.%) enhances formation of the hydration products and compressive strength. Although the hydrated sample having 3 wt.% exhibits the highest hydration products content, it demonstrated compressive strength values lower than those of other HC mixtures and the control sample. These variations in outcomes confirm the fact that the DAS with appropriate Na_2_O content should be prepared to achieve the high performance of one-part HC.

As suggested by Shi et al. [63], the Na_2_O could interact with C-S-H through different three mechanisms. The neutralization of acidic silanol group (Si-OH) is the possible first reaction. The second mechanism includes the partial replacement of Ca ion in C-S-H to yield N-C-S-H. Meanwhile, the third mechanism is the formation of Si—O-Na+ through the complete destruction of the binding capacity of C-S-H. It can be said that these mechanisms mainly depend on the content of Na_2_O in the cement matrix. The first and second mechanisms happen in the presence of relatively low Na_2_O content, whereas the third occurs at the high Na_2_O content. For HC-DAS-1 and HC-DAS-2 mixtures, the appropriate Na_2_O-content leads to the formation of N-C-A-S-H accompanied by compressive strength development. Conversely, a competition between second and third mechanism could happen in HC-DAS-3 mixture, resulting in the formation of hydration products with lower binding capacity compared with those of other samples.

### 3.6. Microstructure

**Figure 10:**
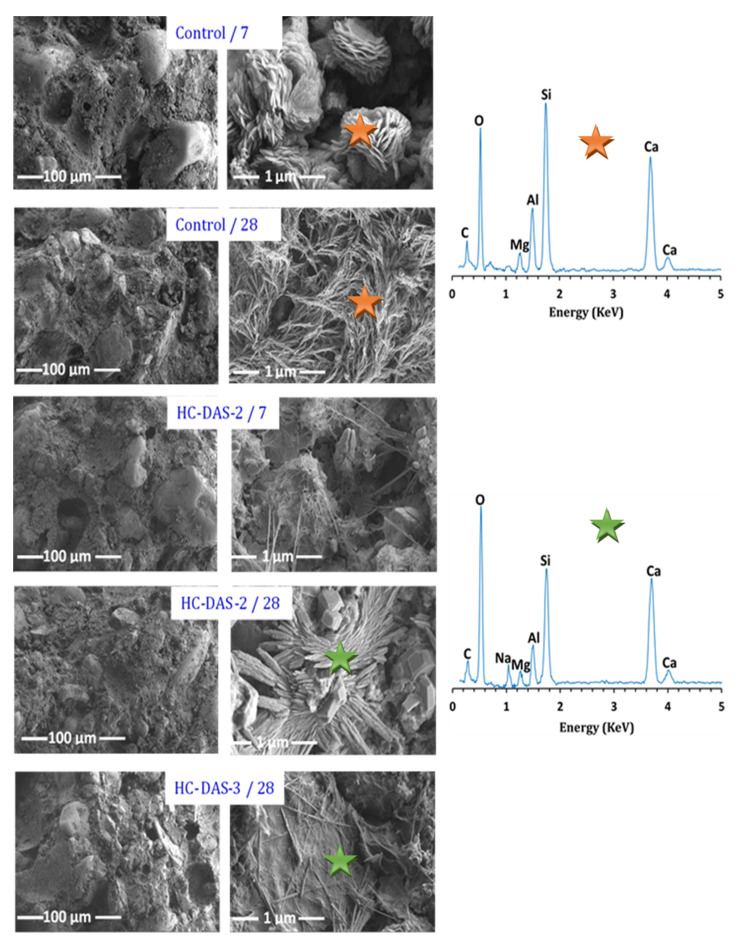
FESEM micrographs of the control sample and HC-DAS-2 mixture at 7 and 28 days of curing as well as the HC-DAS-3 mixture at 28 days of curing.

**Figure 11:**
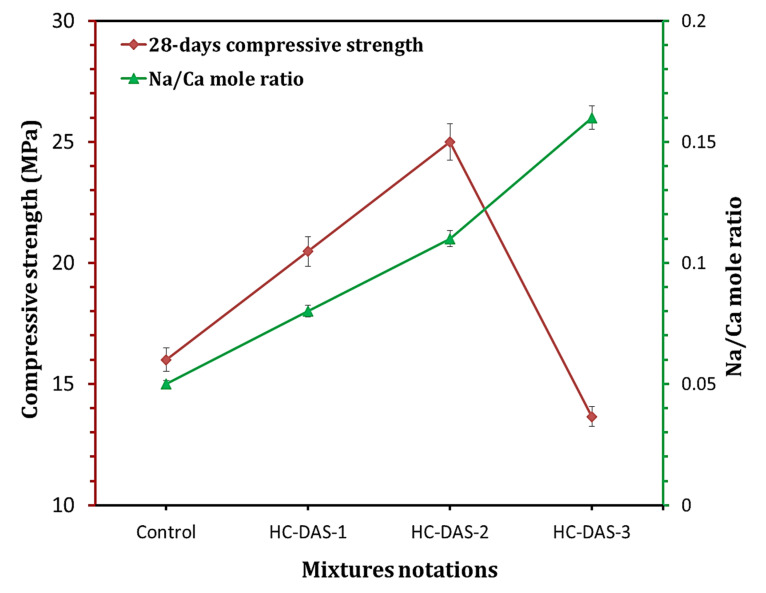
Relationship between Na/Ca ratio and compressive strength of the 28-days hardened mortars having different DAS contents.

Figure 10 displayed the FESEM-micrographs and elemental EDS-patterns of control and one-part HC mortars. For BFS-OPC and HC-DAS-2 mixtures, increasing curing time from 7 up to 28 days enhanced the compaction of microstructure and the formation of binding phases, confirming the compressive strength results. At 7 days of curing, the flakey-shaped phase is the dominant hydration product within BFS-OPC mixture. With time advanced (up to 28 days), this phase transformed to interconnected fiber-shaped phase. The EDS-analysis confirmed that these phases (flakey- and fiber-shaped phases) are mainly affiliated to C-S-H and/or C-A-S-H. No transformation in the morphology of hydration products has been detected with curing time of HC-DAS-2. Nonetheless, increasing curing time caused an enhancement in the formation of needle-C-S-H and rhombohedral zeolitic chabazite (C-N-A-S-H) crystals, as proved by EDS-analysis. This is in line with the XRD results. Additionally, same morphology of chabazite phase has been identified in the previously published works [64,65,66]. Finally, needle-shaped crystals and minor of rhombohedral-chabazite crystals were distributed on gel-like phase within the microstructure of HC-DAS-3 mixture hydrated for 28 days. These variations in the microstructural development and the morphology of the hydration products proved the role of Na_2_O content in the mechanical performance of the prepared one-part HC mortars. Therefore, a relationship between Na/Ca ratio (identified by EDS-analysis) and 28th day compressive strength values of one-part HC mortar was represented in Figure 11. Increasing the NaOH within DAS induces the incorporation of Na into the hydration products. In other words, the replacement of Ca within C-S-H and/or C-A-S-H enhanced with NaOH addition. Interestingly, the best 28th day compressive value was recorded at a Na/Ca mole ratio of 0.11, whereas a significant regression in compressive strength values was achieved when the NaOH content within DAS increased up to 3 wt.%. 

## 4. Conclusions

This paper reported the synthesis and characterization of one-part hybrid cement mortar, in which dry activator–based blast-furnace slag and Portland cement were the main ingredients. Dry activator with different contents were blended with Portland cement to achieve 70 wt.% blast-furnace slag and 1, 2, 3 wt.% NaOH. The hydrated one-part hybrid cement mortar containing 1 and 2 wt.% NaOH was found to exhibit shorter setting time, higher workability, and higher compressive strength compared to blended cement containing the same blast-furnace slag content. Although incorporating dry activator content equivalent to 3 wt.% NaOH accelerated the setting time and enhanced the workability and early compressive strength, it demonstrated the lower compressive strength at later ages of curing. Accordingly, sodium hydroxide content within dry activator played an important role in the performance of the prepared mortar, especially at later ages of hydration. As suggested by the reaction mechanism, dry activator is mainly composed of sodium calcium aluminosilicate species with a high ability to re-interact with water, yielding calcium aluminum silicate hydrate and sodium hydroxide. The liberated sodium hydroxide accelerated the dissolution of unreacted slag particles, resulting in an acceleration in the hydration products formation which coincided with compressive strength improvement. As identified by X-ray diffraction and thermogravemetric analyses, calcium silicate hydrate, calcium aluminum silicate hydrate, and chabazite zeolite are the dominant hydration products with the prepared hybrid cement. It can be said that preparing the dry activator resolved the drawback of the corrosive nature of alkaline solution, which could strongly reflect on the safe use of hybrid cement in different construction projects.

## Figures and Tables

**Table 1:** Chemical oxides compositions (determined by X-ray fluorescence) of the starting raw materials wt.%.

Samples Notations	SiO_2_	CaO	MgO	Fe_2_O_3_	Al_2_O_3_	Na_2_O	K_2_O	Cl	SO_3_	P_2_O_5_	TiO_2_	LOI
OPC	21.01	63.15	2.45	3.36	5.09	0.37	0.07	0.04	2.69	-	-	1.82
BFS	41.51	34.58	4.59	0.57	13.38	1.94	0.86	0.06	1.73	0.32	0.43	-
Sand	94.38	-	-	1.21	2.17	0.12	0.09	0.03	0.09	0.02	0.04	0.13

**Table 2:** Compositions of the prepared dry activator–based slag (DAS).

Dry Activator Notations	BFS	NaOH	Water Content	Theoretical wt of DAS	Actual wt of DAS		NaOH within DAS Powder	BFS within DAS Powder	Combined Water within DAS Powder
Weight (gram)							Wt.%		
DAS-1	100.00	1.00	10.00	111.00	103.18		0.97	96.91	2.11
DAS-2	100.00	2.00	10.00	112.00	105.48		1.90	94.80	3.30
DAS-3	100.00	3.00	10.0	113.00	106.51		2.82	93.88	3.41

**Table 3:** Weights of BFS, OPC, and DAS within one-part HC.

Mixture Notations	BFS	OPC	DAS	Weight of BFS within DAS	Weight of NaOH within DAS
	Weight (gram)				
Control	70.00	30.00	-	70.00	-
HC-DAS-1	-	30.00	72.23	70.00	0.70
HC-DAS-2	-	30.00	73.84	70.00	1.40
HC-DAS-3	-	30.00	74.56	70.00	2.10

## Data Availability

Not applicable.
